# Local solutions, local governance: the municipal health committee initiative in Majdal Anjar, Lebanon

**DOI:** 10.1093/heapol/czag096

**Published:** 2026-08-04

**Authors:** Joanna Khalil, Rouham Yamout, Wesam Mansour, Fouad Fouad, Joanna Raven

**Affiliations:** Department of Epidemiology and Population Health, American University of Beirut, ReBUILD for Resilience, Riad El-Solh, Beirut 1107 2020, Lebanon; Department of Epidemiology and Population Health, American University of Beirut, ReBUILD for Resilience, Riad El-Solh, Beirut 1107 2020, Lebanon; Department of International Public Health, Liverpool School of Tropical Medicine, ReBUILD for Resilience, Pembroke Place, Liverpool L3 5QA, United Kingdom; Department of International Public Health, Liverpool School of Tropical Medicine, ReBUILD for Resilience, Pembroke Place, Liverpool L3 5QA, United Kingdom; Department of International Public Health, Liverpool School of Tropical Medicine, ReBUILD for Resilience, Pembroke Place, Liverpool L3 5QA, United Kingdom

**Keywords:** local governance, health system resilience, fragile and shock-prone settings

## Abstract

In fragile and shock-prone settings, where political instability, economic crises, and institutional fragmentation weaken central authorities, strengthening local health governance has become critical for health system resilience. Lebanon represents an example, having faced compounded shocks including economic collapse, the COVID-19 pandemic, and armed conflict, all of which have strained an already fragmented health system. Despite increasing recognition of the importance of sub-national governance, limited empirical evidence exists on how municipal-level health governance structures function in practice under such conditions. This article evaluates the establishment and functioning of a Municipal Health Committee (MHC) as a pilot model of local health governance, analysing factors influencing good governance practices. Using participatory action research and an implementation research design, qualitative data were collected through process documentation and field observations, six critical reflective meetings, and nine key informant interviews. Data were analysed using the TAPIC framework to examine transparency, accountability, participation, integrity, and capacity in the MHC’s operations. Findings indicate that the MHC enhanced inclusive participation thereby strengthening local legitimacy and trust. The committee progressively embedded transparency and evidence-informed decision-making, including efforts to generate local health data. However, compliance by formulated ideal and institutional integrity was persistently challenged by political interference, patronage networks, weak enforceability of accountability mechanisms, and financial precarity. Sustained mentorship by the research team strengthened governance practices and enhanced the MHC’s ability to implement its mandate effectively. Municipal committees can strengthen equity and resilience in settings through inclusive, evidence-informed processes; however, sustainability depends on local ownership, functional autonomy from political capture, and capacity development.

Key messages

- Local governance structures can strengthen health system resilience and responsiveness in fragile settings.
- Effective decentralization depends on day-to-day governance practices, accountability, and community participation.
- Protecting local governance from political interference is critical for accountable and effective decentralization
- Sustaining decentralized health governance requires institutionalization, capacity strengthening, and strong partnerships.

## Introduction

In low- and middle-income countries (LMICs), good governance is widely regarded as critical for overall growth and development (United Nations 1998). Efforts to enhance good governance practices are not limited to enacting laws, regulations, and administrative procedures. They require actions and mechanisms that enable a society to motivate collective action and implement shared solutions to achieve common objectives (Kaufmann and Kraay 2003). In fragile and shock-prone settings (FASP), good governance shapes the ability of systems not only to absorb shocks but also to adapt and transform in ways that promote equity and long-term resilience (Kruk et al. 2015, Blanchet et al. 2017, Witter et al. 2020).

Governance includes the processes through which individuals and groups organize to achieve collective goals, including evidence-based decision-making, accountability, transparency, resource management, and community participation (Glass and Newig 2019, Seetharam Sridhar et al. 2020). Applied to the health sector, governance involves setting strategic directions, establishing rules and regulations aligned with those directions, allocating resources, and overseeing implementation to ensure effective and equitable service delivery (USAID 2013). These functions can be enacted through decentralization, whereby authority, responsibility, and resources are transferred from central to local levels of the health system. The degree of transferred authority is described as ‘decision space’ (Bossert and Mitchell 2011). In FASP settings where there is a poor economy, institutional fragility, conflict, and violence (Landry et al. 2021), decentralization is often framed as a pragmatic response to weakened central authority. Local actors claim expanded decision space to maintain essential services and respond to local needs. However, decentralization does not automatically generate accountability, integrity, or equity; rather, it reconfigures local power relations and redistributes authority in ways that may either strengthen or undermine governance. To ensure local governance contributes meaningfully to population health outcomes, local authorities must strengthen their capacity to design, implement, monitor, and adapt services to local circumstances (UHC2030 2018). This requires ongoing attention to the principles of transparency, accountability, participation, integrity and capacity, that shape health system resilience (Greer et al. 2016).

Since 2019, multiple crises have broken out in Lebanon with the start of the economic crisis, hyperinflation, and rising poverty and unemployment. Furthermore, the Syrian refugee crisis, which began in 2011, brought around 1.5 million Syrians to Lebanon, adding to the long-standing challenge of hosting roughly half a million Palestinian refugees since 1948 (Rabil 2016, Sumpf et al. 2016). Increased demand for health services overwhelmed the public sector leading to deteriorating health service quality (Yamout et al. 2025). The COVID-19 pandemic further challenged the health system, followed by the 2020 Beirut blast, which destroyed entire neighbourhoods of Beirut including health facilities (Devi 2020), and the 2024 Israel–Lebanon war which exacerbated political and economic instability (Antaby 2024).

Lebanon's health system is heavily fragmented, characterized by a dominant private sector, a massive exodus of health workers, shortage of medicines and supplies, and weakened regulatory role of the Ministry of Public Health (MOPH) (Belayachi 2021, Mjaess et al. 2021, MSF 2021, Yamout et al. 2025). Despite the United Nations High Commissioner for Refugees’ (UNHCR) efforts to coordinate humanitarian aid, equitable access to health services remained challenging (Ammar et al. 2016, APIS Health Consulting Group 2016). In response, an informal health sector emerged, supporting non-governmental organizations (NGOs) in areas with high refugee populations, such as the Beqaa Valley (Honein-AbouHaidar et al. 2019).

ReBUILD for Resilience (ReBUILD) is a multi-country health systems research programme that focuses on strengthening health systems in FASP settings, including in Lebanon. ReBUILD implemented its learning site in the cadaster of Majdal Anjar, Zahle District of the Beqaa Governorate in Lebanon. A cadaster is the smallest unit of territorial administration, delimiting villages in countryside, and neighbourhoods in towns and is ruled either by a municipality (for large cadasters) or by an elected administrator called ‘mokhtar’ (for small villages or towns neighbourhoods) according to UNHCR (2019). This cadaster is ruled by a municipality, and is listed among the 25 most vulnerable cadasters out of 1643 in Lebanon. It hosts one of the country’s largest concentrations of Syrian and Palestine refugees (Interagency Coordination Lebanon 2015).

The Learning Site, which is defined as a medium- to long-term research platform established within a specific geographical area to facilitate collaboration between researchers and local health stakeholders, used participatory action research (PAR) to support the development of a resilient local health system through collaboration with local stakeholders and community-based partners. The aim was to co-develop and implement innovative interventions to deliver inclusive, gender-equitable and quality healthcare, guided by the ReBUILD resilience framework (Kruk et al. 2015, Blanchet et al. 2017, Witter et al. 2020). Two participatory workshops with local partners and stakeholders identified a lack of coordination and planning at the municipal level, leading participants to prioritize the establishment of a Municipal Health Committee (MHC) to govern the local health system as the intervention to be implemented in Majdal Anjar. In Lebanon, formal decentralization policies have designated sub-national health governance roles at governorate and district levels, while municipalities, as elected bodies, are legally mandated to establish committees overseeing local services including health (Lebanese Republic 1977). However, all decentralized structures, either designated or elected operate with constrained decision space and depend on centralized budgets and decision-making (Bossert and Mitchell 2011, Yamout et al. 2025). During acute crises, municipalities temporarily expanded their governance roles, yet reverted to their traditionally marginal position once the crisis subsided, without sustained institutionalization (Gebara 2025, Yamout et al. 2025). Within this context, the MHC functioned as a locally driven governance mechanism that expanded municipal decision space, rather than a formal decentralization reform involving the transfer of health authority from the MOPH to the municipality. It enabled local actors to identify priorities, coordinate stakeholders, mobilize resources, and participate in health-related decision-making within a predominantly centralized health system.

Members’ composition aimed to include health workers from Lebanese, Syrian, and Palestinian communities, women and men, local and international NGOs, civil defence, representatives from other sectors and members from the municipal board. Several municipal board members also served on the MHC, facilitating communication, oversight, and alignment with municipal priorities. The MHC functioned as a complementary governance mechanism within the municipality, maintaining close links with the municipal board while retaining a focus on health-related planning and coordination (more details about MHC establishment can be found in Yamout et al. 2026 Unpublished manuscript). Since its establishment, the MHC has undertaken various initiatives despite limited funding, relying on community engagement, and volunteerism. Activities included breastfeeding awareness campaigns; school health programmes including dental health and first aid training; establishment of a virtual blood bank; medication pooling and redistribution, facilitation of access to free chronic illnesses medications; and targeted plan to improve access for people with disabilities.

Research on humanitarian coordination and health system response during crises widely recognizes the importance of sub-national and community-level governance (Williams 2022, Regmi et al. 2024). However, there is limited evidence on how community-led initiatives, and/or local health governance structures organize healthcare during compounding shocks, particularly in FASP settings, where crises weaken central authorities and exacerbate fragmentation (Truppa et al. 2024). This article addresses this gap by examining how a municipal-level governance structure operates in practice under conditions of compounded crises. It evaluates the establishment and functioning of the MHC as a pilot local health governance model, analysing factors influencing good governance practices and the extent to which these practices contributed to building absorptive, adaptive, and transformative capacities to strengthen local health system resilience in FASP settings.

## Methods

### Study design

The study used implementation research design to track the operations of the MHC in real-world settings (McNulty et al. 2019). Qualitative data were collected through the PAR approach, including process documentation, critical reflective meetings, and key informant interviews.

These data were analysed using the TAPIC governance framework to examine whether and how MHC applied good governance principles to execute its mission, interpreted, when possible, in relation to the ReBUILD resilience framework (Kruk et al. 2015, Blanchet et al. 2017, Witter et al. 2020), drawing on the concepts of absorptive, adaptive, and transformative capacities to situate governance practices within a broader understanding of health system resilience at the local level. Table 1 presents the overall timeline and data sources of the research project, including a summary of the total number of meetings/interviews conducted across the three study components, to illustrate how the different phases of the research were interconnected and contributed to the overall study design.

**Table 1:** Summary of data sources.

Date	Data sources	Description
December 2022	CRM1	Meeting with Learning Site group/Local partners to evaluate the participatory action research approach applied at the learning site leading to the establishment of a municipal health committee (MHC)
January 2023 till May 2025	Process Documentation	Compilation of the MHC meeting minutes since its establishment in January 2023, along with the researchers’ observations and insights, feedback from activities, training, and dissemination events
March 2023	CRM2	Meeting with MHC members to reflect on the concept of decentralization, the strategic plan proposed, the launching event, the planned activities, the initiation of their first community activity ‘breastfeeding training and awareness sessions’
May 2023	CRM3	Meeting with MHC member to evaluate the MHC impact so far on the local health system and next steps
June–July 2023	Key informant Interviews	Nine in-depth interviews with local stakeholders, to explore the factors influencing the development and progress of the MHC.
October 2023	CRM4	Meeting with MHC members to evaluate the dissemination event, where they presented their achievements and the implemented community activities to the public, and reflect on the next steps
April 2024	CRM5	Meeting with MHC members to pause and reflect about the mission and vision of the MHC, its strategic planning, the implementation of the community activities, what can be done differently
September 2024	CRM6	Meeting with embedded research to evaluate how MHC work is aligning with its mission, and to reflect on the difference between MHC role and the NGOs and how to build a solid link between them

### Data sources

#### Process documentation

The Lebanon research team attended MHC meetings regularly totalling 50 times between January 2023 and May 2025. They compiled the meeting minutes electronically, to document what worked, what was blocked, and what facilitated inclusive and equitable approaches, to monitor processes and progress.

In addition, researchers recorded their observations and insights, feedback from activities, training, and dissemination events, along with significant ideas formulated during informal chats. Those notes were compiled electronically in a field journal, outlining the chronological progress of work. The meeting minutes and the field journal were securely stored on a password-protected computer.

#### Critical reflective meetings

Critical reflection meetings (CRM) were designed to facilitate structured discussions with local actors on strategic progress, decision-making processes, and factors facilitating or hindering the MHC's mission for sound local health governance, and for impacts on local health system resilience. The research team facilitated six CRMs between December 2022 and September 2024 at the Municipality of Majdal Anjar (Table 2).

**Table 2:** Critical reflection meetings.

Critical reflective meetings	Date	Number of participants	
		Women	Men
CRM1	December 2022	6	6
CRM2	March 2023	4	6
CRM3	May 2023	4	9
CRM4	October 2023	6	5
CRM5	April 2024	2	1
CRM6	September 2024	6	4

The CRM held in April 2024 was attended by only a few MHC members due to harsh weather conditions in the Bekaa area. Although periodic meetings continued, formal CRMs were suspended due to the security situation, preventing the research team from travelling to Bekaa, with ongoing coordination maintained by a local embedded researcher.

CRMs were guided by an Arabic topic guide and lasted approximately 1 hour (Supplementary Appendix S1). Meetings were recorded, transcribed verbatim in Arabic and translated to English. Transcripts were quality-checked against audio recordings by bilingual researchers to ensure accuracy.

#### Key informant interviews

Nine in-depth interviews were conducted with a purposive sample of local stakeholders, to explore the factors influencing the development and progress of the MHC. Key informants included local leaders, representatives from UNHCR, Palestinian Red Crescent, international NGOs, and local health facilities (Table 3). Purposive sampling was considered appropriate for this study as it enabled the selection of participants with direct involvement in and in-depth knowledge of the local governance dynamics, ensuring rich and contextually relevant insights across the main stakeholder groups involved. While the sample was not intended to be statistically representative, it was designed to capture diverse perspectives from actors central to the intervention and local health governance processes.

**Table 3:** Key informants interviewed.

Respondent code	Organization	Sex	Work location
Kii1	Religious Organization	Man	Zahle
KII2	UNHCR	Woman	Zahle
Kii3 (Phone)	Health Facility	Man	Kfar Danis
KII4	Board of the Municipality of Majdal Anjar/MHC member	Man	Majdal Anjar
KII5	Municipality of Majdal Anjar	Man	Majdal Anjar
KII6 (Phone)	ANERA/MHC member	Woman	Zahleh
KII7 (Phone)	University at Buffalo/Syrian American Medical Society (SAMS)	Man	USA
KII8 (Phone)	Health education/MHC member	Woman	Majdal Anjar
KII9	Al Nasra Hospital for Palestinian refugees	Man	Bar Elias

Interviews were conducted in June and July 2023 by the research team and MHC members, either by phone or in person in the offices of the respective organizations. Interviews followed a topic guide (Supplementary Appendix S2), and lasted approximately 1 hour. Interviews were transcribed verbatim in Arabic and translated into English by a professional translator, with quality checks conducted by bilingual members of the research team.

### Data analysis

J.K. and R.Y. led data collection and formal analysis, while all authors contributed to conceptualization, interpretation of findings, and manuscript review and editing. Purposive sampling, interviews, analysis, and manuscript development were conducted collaboratively by the authors.

The research team used a combined inductive–deductive process (Braun and Clarke 2006). First they collaboratively developed an initial coding framework. First, project documents, narratives of key informants, and field materials were reviewed inductively to identify recurring themes related to the development and functioning of the MHC along with the perception of MHC members and external stakeholders. The first author undertook primary coding using NVivo. Co-authors reviewed coded excerpts, and discrepancies were resolved through iterative discussion to enhance analytical consistency and rigour.

The coded dataset supported multiple analytical streams, with codes subsequently organized according to different analytical frameworks depending on the focus of each study. For the present analysis, codes were interpreted and organized using the TAPIC governance framework (Greer et al. 2019) to examine governance processes and performance. The TAPIC framework was selected because it provided a comprehensive and practical approach for analysing governance in health systems through five interconnected dimensions particularly relevant to decentralized and community-based governance in fragile settings. The TAPIC constructs were operationalized as follows: Transparency examines whether decisions-making processes are visible and accessible to the community in order to build trust and reduce corruption. Accountability analyses the mechanisms that clarify the roles and responsibilities of members to ensure responsibility for their engagement. Participation assesses the inclusiveness of the MHC model and the extent of engagement of external stakeholders and community members in planning and decision-making. Integrity examines organizational structures and ethical practices to identify how conflicts of interest and inefficiencies are managed. Capacity examines the availability of resources, skills and information needed for the MHC to fulfil its declared functions.

The entire research team participated in reflective discussions to refine emerging themes and ensure analytical rigour, coherence, and accuracy.

### Ethics

Ethical approval for this study was granted by the Institutional Review Boards of the authors’ institutes.

Written informed consent was obtained from participants interviewed in person and for the CRMs. For phone interviews, consent form was shared electronically via WhatsApp and participants provided written consent by replying with a confirmation message. Permission to record interviews was obtained prior to data collection.

## Results

The findings analyse both enabling practices and persistent challenges shaping MHC governance practice. Table 4 summarizes the findings of the study under the TAPIC Framework.

**Table 4:** Summary of findings using TAPIC framework.

TAPIC framework	Successful actions	Challenges
Transparency	Shifting attitudes towards transparency, openness, accountability, and public trust Transparency in communication to convey MHC’s objectives and functions Transparency features dominate the MHC's strategic plan	Fractured trust between the community and the municipality Debate surrounding the anonymity of MHC members Instances of breach of transparency Procedural transparency can be fragile when clientelism incentives are present
Accountability	Embedded researcher to coordinate, organize and oversee the MHC day-to-day/Aiming to formalize the researcher’s position permanent municipal employee Membership request form for prospective members Regular reflective and self-evaluation meetings were to allow members to articulate their perception of the MHC’s mission and their roles	Interference form political parties/powerful families seeking control over resources Major obstacle to autonomous decision-making Unproductive partnerships with NGOs not aligned with the MHC’s strategic goals Tendency towards individual work rather than teamwork and limited collaboration Absenteeism and persistent operational failures, undermined institutional coherence Failed to establish institutional authority to enforce commitments
Participation	Shift from authoritative, middle-aged to include women, younger activists, and, crucially, the Syrian and Palestinian health workers Viable path to building trust and enabling collaboration/succeeded in developing initiatives that actually mattered	Atmosphere of hostility towards the refugee population No women participation in the municipality board
Integrity	MHC affiliation with the Municipality as a ‘Trust Seal’ MHC president negotiated operational autonomy without undue political interference Restriction of meetings to an ‘executive committee’ of active members Appointment of an embedded researcher to oversee daily operations	The culture of patronage and clientelism undermined institutional integrity of the MHC Risk of MHC being associated with perceived municipality corruption
Capacity	MHC benefits from members with diverse backgrounds in Public Health, Business Administration, and other relevant topics members towards evidence-based governance Collaboration with research consortium and academia Data collection as a transformative approach to respond to population needs Leveraging volunteerism MHC initiated independent data collection Strategic utilization of existing resources Exploring alternative funding sources	Lack of data/Unsupported evidence Data-sharing barriers Financial constraint and limited resources

### Transparency, amidst traditions of opacity and clientelism

Transparency emerged as the first governance challenge in the MHC's early formation, reflecting the fractured trust between the community and the municipality. As one MHC member remarked: ‘Do not expect support from the community. Long ago, the community lost trust in the Municipality, and everyone is seeking alternative means to access the services they require’ (MHC Weekly meeting). Consequently, the primary task was to establish transparent communication to convey the MHC’s objectives and functions effectively to the community.

Some members, like the Syrian MHC members experienced in humanitarian programs, advocated transparent practices to enable trust and institutional evolution; whereas others maintained the persistent culture of opacity. For example, during an early meeting, a participant openly suggested manipulating population figures to get more resources, and no one considered this suggestion unacceptable.

Over time, through mentorship, workshops, and discussions, the MHC formally adopted transparency as a core value in its strategic plan. However, some members still struggled to translate the principle into practice. For example, an MHC member retained donated medications at home, perhaps with the intention of distributing them later to boost community loyalty ahead of a future election. This incident exposed how fragile procedural transparency can be when clientelism incentives are present.

An interesting moment for institutional transparency occurred during the debate over member anonymity. Some argued anonymity would protect members from solicitation; others insisted that good practice required the disclosure of their identity. Finally, influenced by the experience from the ReBUILD programme in Nepal, where health committee members’ details and photos are publicly displayed in all health facilities, the MHC chose to disclose all identities on social media. As the MHC president highlighted, ‘I believe that we all should be known to the public. It is better for our credibility and community trust’ (CRM4).

The concern of being solicited revealed a tension: both the community members and some of the MHC members perceived the MHC as a conduit for aid, rather than as a health governance body. MHC members gradually realized that transparent communication of the MHC governance mandate is necessary to manage community expectations. As one member stressed, ‘We should clearly communicate that the MHC is an entity enabling health management and be explicit about our strategic goals’ (CRM4). Public dissemination of the strategic plan marked a critical shift towards transparent communication, clarifying what the MHC could and could not provide: ‘People became aware of our work and understood the benefits they could get from it’ (CRM2).

This represented an early expression of transformative capacity, as the MHC sought to institutionalize transparency as a governing norm in a context historically shaped by opacity and clientelism, though the fragility of this shift, illustrated by persistent breaches, signals that transformation remained incomplete.

### Accountability, amongst external pressures and institutional fragility

#### External pressures

Within the local power landscape, the MHC struggled to realize its compliance by its public mandate. MHC members anticipated interference from municipal board members affiliated with political parties or powerful families seeking control over resources. This interference was perceived as a major obstacle to autonomous decision-making, as one member expressed: ‘If MHC undertakes all that is planned, it will be successful [….] and this will solve most of the health issues in the village. But the question is: would it be allowed to be successful? Will the proper conditions be created for it to act?’ (CRM1).

Financial accountability was similarly limited. The ‘local governance model’ failed to convince local funders, while external funders imposed their own program priorities rather than abiding by MHC requests. As one member explained, ‘At the end of the day, the MHC may find itself unable to compete with other funders, who will impose their programmes and recruit the providers they want, regardless of our opinion’ (MHC Weekly Meeting).

#### Internal accountability crisis

Some of the broader social dynamics in Lebanon, such as a tendency towards individual work rather than teamwork and limited collaboration, were also reflected within the MHC, contributing to scepticism towards collective action and creating challenges in enforcing agreed rules. Some members joined MHC seeking personal benefit rather than community service. As one municipal board member acknowledged ‘We have to face it; we are attracting people looking for easy pay, rather than people committed to serving their community’ (KII4).

Furthermore, opportunistic figures appeared to grab benefits, such as securing training certificates without attendance or favouring relatives for positions, and bypassing the committee to pursue external projects independently, monopolizing benefits and hindering opportunities for MHC intervention.

Despite researchers’ efforts to boost internal accountability, absenteeism and persistent operational failures, including poor meeting minutes, and delayed or incomplete task execution further undermined institutional coherence. During a reflection meeting, a frustrated member noted, ‘We are always facing the same problem… We cannot continue like that. We have to shake things up’ (CRM4).

Furthermore, the MHC failed to establish institutional authority to enforce commitments or sanction members who did not fulfil their agreed responsibilities. Although members occasionally exerted peer pressure on those who failed to meet their obligations, these efforts remained interpersonal rather than organizational.

Even the mayor delegated enforcement authority to researchers rather than taking disciplinary measures: ‘I confer upon you the authority to evaluate the candidates… and avoid any future rumours about me backing a health committee whose members are not fully committed’ (CRM1).

#### Building accountability

Recognizing these challenges, the research team supported the development of administrative accountability mechanisms: (i) An embedded researcher was appointed as coordinator to track commitments, and ensure operational follow-through; (ii) the team encouraged the MHC to negotiate with the municipality to formalize the researcher’s position as a permanent municipal employee, ensuring sustainable institutional oversight; (iii) the team facilitated self-evaluation sessions for structured peer accountability; (iv) To mitigate opportunism, the MHC introduced a membership form mandating voluntary service commitment and restricting the political participation in specific meetings; (v) regular reflective meetings were conducted to allow members to articulate their perception of the MHC’s mission and of their role within it. These measures reflected adaptive capacity, as they introduced new governance practices and accountability mechanisms to address operational challenges and strengthen committee functioning. However, accountability mechanisms remained researcher-dependent rather than fully institutionalized within the MHC, limiting their transformative potential and threatening sustainability once the PAR process ends.

### Participation

When the research team first encountered the municipality, they found a homogenous circle of authoritative, middle-aged businessmen from the two dominant families who held a monopoly over the town’s affairs. These two families had formed a tactical alliance to win the municipal elections, effectively excluding the rest of the residents. Breaking this entrenched elitism required more than workshops; the team engaged in months of negotiations to open up the MHC membership to a broader population. Progressively, the table grew to include women, younger activists, and, crucially, the Syrian and Palestinian health workers who had previously been treated as outsiders despite being the backbone of the town’s healthcare. This transition functioned as an opening of a previously closed system, while boosting the MHC’s effectiveness.

This reflected transformative capacity, as it altered established power relations and governance arrangements by broadening participation, redistributing influence, and creating more inclusive decision-making structures. For both MHC members and the key informants interviewed, this inclusiveness was seen as a viable path to building trust and enabling collaboration. By bringing in different perspectives and exploring the needs of all social categories, the MHC succeeded in developing initiatives that actually mattered. Projects like the virtual blood bank and the chronic medications plan worked because they were designed by people exposed to the realistic, often adverse conditions of health delivery. In the end, this inclusivity gave the MHC the legitimacy to operate; it forced the elite to resign their exclusivity over every decision in favour of a real community leadership that transcended socio-political divides.

The inclusion of Syrian refugee doctors was a major turning point, occurring within an atmosphere of hostility towards the refugee population. Bringing Syrian professionals to the table was also a powerful message that the host and refugee communities could unite their expertise and assets for the collective welfare (CRM4). Less than a month after their inclusion, one Syrian doctor was elected as the MHC vice-president. The MHC president noted ‘He did an excellent job. We should thank him. We should also thank the committee’s female members’ (CRM4).

As the president’s remark suggests, the inclusion of women challenged their normative exclusion from local leadership (there are currently no women on the municipality board). Their involvement transcended symbolic participation; women became central to the operational activities, establishing a precedent for gender-responsive governance. This success encouraged other women to join, proving that their leadership was an added value rather than a formality.

Finally, after an initial period of hesitation, the committee saw an influx of individuals from opposing political factions. Encouraged by the MHC’s discourse of transcending partisan divides, MHC positioned itself as a representative governing body rather than an extension of the dominant political faction. As one member emphasized, ‘Even if you do not agree politically with the municipality, you have your place in the MHC. After all, the Municipality is for all its community, even those who did not elect the board’ (CRM2).

### Integrity

The creation of a local structure, affiliated with the elected local authority, required a delicate balance between institutional affiliation and functional autonomy. The dominant culture of patronage and clientelism, alongside opaque and renegotiated local power dynamics, undermined institutional integrity by compromising the MHC’s coherence with its stated mission.

Since inception, the mayor and the municipal board have shown their support through active involvement in MHC deliberations and dissemination events. MHC members viewed the municipality as a ‘Trust Seal’, recognizing it as a source of credibility and legal compliance (CRM1). External stakeholders echoed this opinion, noting that the MHC affiliation with the municipality provided both the ‘necessary connections’ (KII2) and the security required to exert community action (KII4). However, this privileged position also increased exposure to the dominant patronizing practices.

CRMs and field notes captured the growing concern about balancing the need for legal backing of MHC work by the municipality and the risk of being associated with perceived municipality corruption. Members expressed concern that their operational freedom would be ‘dangerously contingent’ on political priorities rather than health needs (CRM4). These concerns were confirmed by the pervasive patronage practices they observed. During the strategic planning workshop, a municipal board member argued that the MHC must ‘maintain a power gradient’ over NGOs and partners to force their priorities.

Interference from ‘political members’ extended beyond simple advice; influencing project implementation and resource allocation, for their own private interests. ‘Technical’ members were then required to defend the scientific logic of their plans against political impulses. This contributed to members’ frustration; and in some cases, the withdrawal of skilled volunteers. One community volunteer resigned the moment they realized the health educators were appointed based on personal preference of board members rather than merit (KII6). While some MHC members resisted this intrusion, others accommodated themselves by forming alliances with political interveners to benefit from the outcomes of their interventions.

Consequently, during the first 2 years, the MHC struggled with hierarchical control more than with managing its work effectively. As one external stakeholder reflected, without full knowledge of the internal dynamics of the MHC, decentralization could be the solution, but if it reproduces corruption of the central level, it will yield the same failure. He noted explicitly ‘[…] we need to get rid of the mentality of patronage and clientelism… that is ruining all the technical efforts’ (KII9).

To safeguard institutional integrity, the MHC president negotiated operational autonomy, to be able to implement day-to-day decisions independently, without undue political interference. He also restricted the meetings to an ‘executive committee’ comprising only members with professional expertise in the social sciences, community work and evidence-based decision-making, who were actively involved in MHC meetings and activities. In addition, the research team appointed an embedded researcher to coordinate and oversee daily operations and ensure alignment with the MHC mission. These measures enabled functional internal scrutiny, and blocked clientelist behaviour. These actions reflected adaptive capacity, as the MHC introduced new governance arrangements and oversight mechanisms to protect its functioning and integrity in a politically sensitive environment. However, because these safeguards remained dependent on individual actors rather than institutionalized rules and structures, their transformative potential remained limited, and the transition towards fully professionalized governance was not achieved.

### Capacity

#### Access to timely and reliable information

The first obstacle was the lack of data, as population estimates varied widely and numbers regarding disease prevalence were not based on evidence. Municipality board members would present unsupported evidence, such as, ‘we have in the village the highest prevalence of diabetes in Lebanon’ or ‘the majority of youth are drug addicts.’ Even the UNHCR could not resolve access to information: ‘Several studies have been conducted by partners but we do not have any central repository to present the results of all those studies’ (KII2).

In this context, the MHC moved towards evidence-based governance. Academically trained members developed local data collection tools, and initiated plans for a community-level health assessment for which MHC raised funds. MHC members viewed data collection as a transformative approach to respond to population needs. As one member put it, ‘With this data, we will not just guess anymore. We will know exactly what diseases are hitting our streets and can tell the dispensaries exactly which patients need the most help’ (KII4). This reflected transformative capacity, as the MHC sought to institutionalize evidence-informed decision-making and establish new ways of governing local health priorities. However, this enthusiasm was constrained by data-sharing barriers. MoPH, UNHCR, universities, and NGOs, did not share data with the MHC, citing ‘confidentiality protocols.’ In response, MHC initiated independent data collection, including documenting participants of awareness campaigns; recording children participating in school health programmes; and targeted surveys. In addition, they also explored data-sharing agreements as a mechanism for NGO collaboration. These responses reflected adaptive capacity, enabling the MHC to continue generating and using evidence despite restricted access to existing data systems.

#### Access to financial and human capital

Although the Mayor promised to allocate funds for MHC operation and employment of experts, this did not materialize, owing to the economic instability of the country following the major collapse and the armed conflicts (KII5). MHC mitigated this shortfall by mobilizing existing expertise and exploring alternative sources of funding.

Volunteerism, inspired by the work of crisis cells during COVID-19, became central to the MHC operations. The Mayor further pushed the MHC in this direction: ‘we need a cohesive team of professionals and young volunteers working closely with the community’ (KII5).

Reaching out to local businesses to raise funds was another idea, however after informal inquiries met with little interest, they decided to postpone until they could ‘demonstrate key achievements to deserve such support.’ (CRM2).

In addition to the embedded researcher, whose role was remunerated through support from the research team, and the AUB researchers who provided mentorship and training, MHC operations relied on the limited resources available for PAR, covering transportation and communication costs. The remaining work including training, capacity building, fieldwork, data collection, and reporting was provided pro-bono by MHC members. These actions reflected primarily absorptive capacity, as the MHC sustained its core functions despite severe resource constraints by relying on volunteerism, existing expertise, and external support.

#### Access to competence and expertise

Despite financial limitations, the MHC demonstrated internal competence, benefiting from a membership with diverse backgrounds in public health, business administration, and clinical practice. For example, the first aid training was delivered by a nurse, who had volunteered for many years with the Red Cross, while a social worker working from a care centre for children with special needs provided an orientation on disabilities. Capacity building activities including training on equity, gender, strategic planning, and various public health topics were supported by the ReBUILD research team. These additional training and mentorship further strengthened members’ capabilities, demonstrating adaptive efforts to enhance the committee's effectiveness within existing structures.

## Discussion

This article examines the operations of the MHC as a pilot model of local health governance, exploring the factors that shape effective governance practices using the TAPIC framework (Greer et al. 2019). The MHC initiative aimed at operationalizing decentralization by establishing a municipality-based committee to govern health at the local level. In contexts of multiple crises and weakened central governance, to what extent can municipality-based health committees contribute to strengthening local health governance and system resilience? Siddiqi et al. (2009) argued that local governance of the health system can be strengthened irrespective of a country’s broader governance environment. Local governance mechanisms, such as health boards and community committees may enhance health system resilience by filling gaps left by weak governments and ensuring that decisions reflect local priorities (Abimbola et al. 2016).

Here lies the role of local actors in governance, including the participation of the public sector, civil society and the private sector in ensuring population health. This was reflected in the inclusive MHC composition, consisting of local health professionals from Lebanese, Palestinian and Syrian communities, as well as local and international NGOs, municipal board members and representatives from other sectors (education, environment, civil defence), enabling more context-responsive decision-making.

Local governance entails shifting the understanding of health policy and governance from a simple top-down model to a cyclical process integrating bottom-up and top-down interactions. Governance should no longer be seen solely as a national-level function but as a ‘multi-level’ and ‘polycentric’ system (Bigdeli et al. 2017). This perspective emphasizes the role of local actors in shaping public policies and highlights the importance of horizontal policy networks that connect actors across organizations to collaborate, learn and act (Wagenaar and Cook 2003, Laws and Hajer 2006), particularly through the PAR approach adapted in Majdal Anjar.

However, interference from local authorities affiliated with political parties presents a significant challenge for governance reforms, transparency, and accountability. Decision-making within local governance structures like the MHC was influenced by political, economic, and institutional challenges. Power struggles and opposition from entrenched interests hindered the MHC’s progress. Consistent with literature on decentralization, reforms can leave systems vulnerable to elite capture and corruption when local officials exercise increased authority without adequate accountability mechanisms, weakening transparency and slowing governance reform (Zarychta et al. 2024).

Research on health facility committees in LMICs similarly highlights how local politicians and elites can shape priority setting, and manipulate membership to serve vested interests, undermining transparency and performance (e.g. through political patronage and interference). The MHC experience shows this tension: increased local decision space did not automatically translate into enforceable accountability. Rather, it expanded room for local action while exposing the MHC to political interference, patronage, and weak enforceability. To uphold integrity, the MHC sought to establish legitimacy through consistent engagement with both municipality and community, while directly confronting patronage, political favouritism, and erosion of trust (McCoy et al. 2012).

Governance in LMIC health systems is rooted in everyday practices (Gilson et al. 2017). This paper scrutinized the step-by-step evolution of the MHC, situating governance within routine decision-making processes and daily interactions. Such an approach moves beyond viewing governance as a predefined system objective (Gilson et al. 2017, Pyone et al. 2017) and highlights how transparency, accountability, and commitment to public welfare are actively produced through ongoing deliberations and organizational behaviours. In this sense, the study contributes empirical insight into how governance principles are not simply designed institutionally but negotiated and enacted through day-to-day practice in fragile settings.

Building on this perspective, the research team’s continuous engagement with the MHC fostered a deliberate shift towards assuming local governance responsibilities, embedding transparency as a core strategic value guiding decision-making. Structured discussions, community-oriented events and systematic oversight of daily operations strengthened internal accountability, reinforcing the MHC’s commitment to public welfare. However, these accountability gains still relied partly on external support, leaving questions about their long-term sustainability, suggesting that local governance capacity may require sustained facilitation before it becomes institutionally embedded.

In examining how decentralization influences health system equity, efficiency and resilience, Abimbola et al. (2019) explained the ‘Close to Ground’ decentralization trigger mechanism. Governing ‘close to the ground’ can enhance equity by tailoring decisions to local needs. The MHC’s evolving commitment to evidence-based governance reflects this approach. Through transparency, capacity building and community engagement, the MHC integrated evidence generation and utilization into its strategic goals and daily operations. Surveys, awareness sessions, and local data collection sought to ensure its programs’ alignment with local community needs.

However, limited centralized resources available to municipalities and reliance on ad hoc evidence generation by external organizations limited the MHC efforts. In response, MHC members expressed a strong desire for self-sufficiency in evidence generation, advocating for capacity building to strengthen data collection and analysis. Central to this vision is the establishment of a comprehensive, localized health database to inform targeted interventions. This aligns with evidence from LMICs where decentralized, locally owned databases enhance the relevance, timeliness and use of evidence in decision-making, while strengthening accountability and responsiveness to population needs (WHO 2010).

Financial limitations further shaped governance performance. Limited municipal resources and irregular funding prompted the MHC reliance on volunteerism and member expertise while identifying alternative funding sources. Such findings resonate with decentralization literature showing that local governance amid financial uncertainty often necessitates flexible and context-sensitive responses that combine formal resources with informal mechanisms, such as social capital and community engagement (Bossert and Mitchell 2011). Importantly, this body of work emphasizes that long-term sustainability cannot rely solely on short-term project funding, but rather depends on the strategic institutionalization of local governance structures, participatory approaches rooted in community needs, and adaptive capacity to navigate financial and organizational constraints.

### Implications for policy and practice

The experience of the MHC in Majdal Anjar underscores significant implications for decentralized health governance in fragile settings. First, local actors can shape health system governance, where national-level institutions are weakened by political and economic crises. By establishing a community-based committee and adopting evidence-informed decision-making processes, the MHC exemplifies how local governance structures can enhance health system responsiveness and resilience, while fostering transparency and accountability. Second, decentralization requires protection against elite capture and mechanisms for enforceable accountability, not only increased decision space. Supporting local health governance therefore includes more than relying on decentralized authority, and requires capacity building, systematic data collection, institutional oversight and integration with municipal structures. Practically, embedding PAR, continuous training, and community engagement can enhance local ownership and sustainability of interventions. However, political interference, resource constraints, and limited centralized support can undermine local initiatives, necessitating strategies that protect committee autonomy while fostering collaboration with state and non-state actors.

Future research should examine how municipal health committees can be institutionalized and sustained beyond externally supported initiatives, and assess their long-term effects on health system governance and resilience across diverse fragile settings. Comparative studies are also needed to identify the governance arrangements and accountability mechanisms that enable local committees to expand decision space while safeguarding integrity, equity, and evidence-informed decision-making under conditions of protracted crisis.

### Strengths and limitations

This study benefits from a longitudinal participatory design spanning over 2 years, which enabled close observation of the development and implementation of an innovative model for decentralized health governance through the MHC in Majdal Anjar, which brought together local community members, including refugees, to support evidence-informed, transparent, and inclusive decision-making. The study also advances the application of PAR in FASP by demonstrating how community-led governance initiatives can strengthen health system resilience and responsiveness in contexts affected by exclusion and multiple vulnerabilities. The integration of multiple formal and informal qualitative data sources strengthened data triangulation and increased the credibility and robustness of the findings.

While the study spanned over 2 years and enabled in-depth observation of implementation processes, this duration was insufficient to capture longer-term community-level health outcomes and sustained governance impacts. The study’s context-specific focus on a pilot Municipal Health Committee in Majdal Anjar limits the transferability of findings to other settings, where differing socio-political dynamics, NGO coordination structures, and local infrastructure may shape governance processes differently. In addition, periods of interruption caused by power dynamics, political sensitivities, and resource constraints limited the assessment of longer-term outcomes and the continuity of the intervention.

While the study was conducted between 2022 and 2024, the governance challenges examined remain highly relevant due to the persistent political, economic, and institutional crises affecting Lebanon’s health system, which continue to shape decentralized and community-led responses.

## Conclusion

This study demonstrates that municipal-level health governance structures can strengthen local health system resilience in FASP. The experience of the Municipal Health Committee in Majdal Anjar illustrates that participatory and evidence-informed governance can emerge locally and progressively institutionalize principles of transparency, accountability, participation, integrity and capacity. Through sustained engagement, mentorship, and structured reflection, the MHC evolved from a loosely coordinated initiative into a recognized local governance actor capable of mobilizing community trust, fostering cross-sectoral collaboration, and initiating context-responsive interventions. However, decentralization alone does not guarantee good governance. Political interference, patronage networks, weak enforceability of internal accountability mechanisms, and financial precarity posed continuous threats to institutional autonomy and integrity. Local health governance in FASP settings is therefore not only a technical reform but a negotiated political and social process. The study shows that municipal committees can expand local decision space and strengthen responsiveness, but that their effectiveness depends on how accountability, integrity, and evidence generation are institutionalized under conditions of fragility. Strengthening municipal governance demands deliberate investments in local capacity, embedded accountability mechanisms, and protected decision space, alongside policies that foster polycentric and community-centred governance. When supported appropriately, local committees such as the MHC can become viable platforms for advancing equity, resilience, and public welfare in contexts of protracted crises.

## Supplementary Material

czag096_Supplementary_Data

## Data Availability

The data underlying this article will be shared on reasonable request to the corresponding author.

## Reflexivity statement

Our multidisciplinary team included researchers from the American University of Beirut based in Lebanon and the Liverpool School of Tropical Medicine in the United Kingdom, with expertise in public health, health systems research and community engagement. The authors represented women and men of different ages, national backgrounds, and professional seniority, ranging from early-career researchers to senior academics. The Lebanon-based team, are bilingual and familiar with the socio-political context of the community, which facilitated communication, cultural sensitivity, and participant comfort during fieldwork. Collaboration across institutions and contexts brought diverse disciplinary and experiential perspectives that enriched the study design, analysis, and interpretation. Throughout the research process, we held regular reflexive discussions to examine how our positionalities and relationships with municipal actors and community members shaped access, power dynamics, and the framing of findings.
