# Supplementary material for: Local solutions, local governance: the municipal health committee initiative in Majdal Anjar, Lebanon
Source: Health Policy Plan. 2026 Aug 4;41(8):1461–72. doi: 10.1093/heapol/czag096 (PMC13586627; doi:10.1093/heapol/czag096)
Supplement: Supplementary file 1 [file czag096_supplementary_data.zip › MHC paper - Appendix 1.docx]

# Appendix 1: Critical Reflection Meeting Guidelines

**Purpose**: to support collective reflection to identify (1) how the context is changing in the site over the past period (e.g. month or at maximum quarter); (2) how the innovations are being implemented or adapted – what the challenges have been, the factors, processes and initiatives that facilitate or hinder implementation; (3) the effects of the innovations on the local health system and its equity; and (4) how the actions may need to be altered to be more effective in the next period.

**Attendees:** Lebanon team; community-based partners; research support network engaged in leading on intervention/actions.

**Time:** 1 hour

**Discussion questions**:

1. What important changes have occurred in the local health system over the past month/since we last met?

a. How have they affected the system performance, equity, and resilience?
b. What/who drove those changes?
c. What do they imply for the next period and how do we act at the local level?

2. How is implementation of the agreed priority action progressing?

a. How far has it been enacted? Why/why not?
b. If it has been adapted, how and why?
c. What are the barriers and facilitators for it?

3. What effects has it had to date?

a. Are we seeing the anticipated benefits? If not, why not?
b. Any unanticipated effects, also for other areas of the system and its resilience?

4. What does this mean for future engagement?

a. How should we change out approach to be more effective?
b. Who should we focus on engaging to bring the needed resources?
c. What have we learned from our PAR process to date? What could be improved?

**Output:** There should be a structured note of any significant discussion points, including areas of uncertainty or consensus, and decisions about strategy changes for the next period.
