# Supplementary material for: Local solutions, local governance: the municipal health committee initiative in Majdal Anjar, Lebanon
Source: Health Policy Plan. 2026 Aug 4;41(8):1461–72. doi: 10.1093/heapol/czag096 (PMC13586627; doi:10.1093/heapol/czag096)
Supplement: Supplementary file 1 [file czag096_supplementary_data.zip › MHC paper - Appendix 2.docx]

# Appendix 2: Key Informant Interview topic guide at end of cycle

**Purpose**: to understand the perspective of a range of engaged or affected stakeholders in the local health system on the participatory action cycle and its effects. These questions will need to be adapted according to the actions which are prioritised, and probes added as appropriate during interviews.

**Questions**

After explaining the background of the project and the purpose of the interview, informed consent will be obtained.

**1. Respondent’s role**

What has been your role in this area? How long have you been in it? Were you engaged in any of the action research? If so, how and why?

**2. Rationale for actions**

What do you see as the greatest challenges in terms of resilience in the local health system? What would you prioritise for engagement?

Are you aware of recent attempts to strengthen the health system? Do you think they were targeting an important aspect with potential to improve equitable services locally?

**3. Experience of actions**

How do you think the actions were carried out? What were the challenges, and why? Any successes (and why)?

**4. Effects of actions**

Did you notice any changes which were brought about by these actions (changes that were planned or unplanned)? What were they, and who did they affect? How did any context or wider changes affect them?

**5. Lessons and recommendations**

What lessons would you draw from this last period?

What do you think is important and possible to change over the next few months to bring about a fairer, more effective and more resilient health system in your area?

Who should be involved and how?

What might your role in these actions be?

What have you learned or gained, if anything, from being part of this learning cycle to date? Do you have any suggestions for how it could be improved?
